# Pulsed radiofrequency as alternative method for phantom pain treatment. Case report

**DOI:** 10.1002/ccr3.3110

**Published:** 2020-07-20

**Authors:** Krzysztof Brzeziński, Anna Renata Rękas‐Dudziak, Agnieszka Maruszewska

**Affiliations:** ^1^ Institute of Rural Health Lublin Pain Practice Lublin Poland

**Keywords:** iliac nerve, Phantom pain, pulsed radio frequency, sciatic nerve

## Abstract

Pulse radiofrequency is a safe method of fighting phantom pain. It creates the possibility of treating cases that have exhausted other therapeutic options.

## Graphical abstract

Pulse radiofrequency is a safe method of fighting phantom pain. It creates the possibility of treating cases that have exhausted othr therapeutic options.

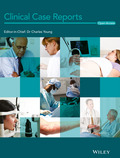

## 1 CASE PRESENTATION

Phantom pain mainly affects patients that have undergone limb amputation and mostly appears only within the first months after surgery. It usually disappears after prostheses have been implanted, when the brain receives information that the amputated limb, which is effectively “mimicked” by the prosthesis, has returned to its place and thus the disease process stabilizes. In some patients, however, the pain may be chronic and persist up to several years. There are also cases of recurrence of pain after a period of complete resolution. Recurrent pain is reported by 60%‐80% of patients ^1^, ^2^, ^3^, regardless of the cause of the amputation: trauma or chronic diseases. Treatment of phantom pain is a challenge to the attending physician. Due to the complex nature of this type of pain, there is no exclusive treatment scheme, which should always be established taking into account individual needs.

Similarly, there is also no universal way of treatment. An effective phantom pain therapy should include so‐called combination pharmacotherapy, that is, the use of analgesic and antidepressant drugs, invasive methods, psychotherapy, and rehabilitation.

The patient described here underwent amputation surgery at 1/3 of the thigh due to critical ischemia of the left lower limb. The critical ischemia that preceded amputation resulted from chronic diseases patient had. These are as follows: hypertension, type 1 diabetes, glycosylated hemoglobin over 8, 5 mg%, and hypercholesterolemia. The patient was taking oral medication and insulin for long period of time, but the level of atherosclerosis was so advanced that it caused limb ischemia and the result was amputation. Before the amputation procedure in the hospital's vascular surgery department, the artery was unclogged, but with no result. After 4 days of surgery, amputation was performed.

Three months after the surgery, she presented with stump pain and phantom pain. During the appointment at the pain management clinic, the patient described the sensation in the lower left limb as burning and searing pain, accompanied by numbness of the stump and phantom pain of the amputated shank and foot. The previous treatment of the patient with NSAIDs and Tramadol at the daily dose of 200 mg proved ineffective. The pain with an intensity of 6‐8 on the NRS scale (0‐10) caused disruption of sleep 3‐4 times per night. MRI examination of the stump revealed absence of a neuroma in the operated area.

Physical examination performed after interviewing the patient showed hyperalgesia of the medial region and allodynia of the lateral and posterior regions of the stump. Trigger points were located in the groin area and on the lateral and medial side of the amputated limb. Pharmacological treatment with 25 mg/day amitriptyline, 900 mg/day gabapentin, and 20 mg/day oxycodone was introduced. Reduction in daytime pain to NRS 3‐5 was achieved, whereas the night pain was unchanged. This prompted a search for a more effective method for alleviation of the chronic pain to ensure patient comfort. Invasive treatment was proposed and implemented after obtaining full informed consent from the patient. The condition form performing the PRF procedure was a positive response to the regional blockade.This is the criterion for qualifying for the PRF procedure. Pain decreased significantly on the NRS scale for 4 weeks. This allowed the patient to be qualified for PRF procedure.

The procedures were performed in sterile conditions in compliance with the principles of aseptics. An ultrasound device (eZono 4,000) was used to visualize the needle. Three trigger points were blocked by administration of 3 mL of 1% lidocaine and 4 mg of dexamethasone in each point using a spinal needle (22G 75 mm Engeman). After blocking, the NRS daytime pain intensity declined to 2‐4 points. Unfortunately, the procedure did not reduce the nighttime and phantom pain. Due to the low efficacy of the treatment, after a week, ultrasound‐guided femoral nerve blockade through anterolateral access and sciatic nerve blockade through Labat access were performed by administration of 5 mL of the solution described above. The treatment resulted in complete relief of nighttime symptoms. Given the good results of the invasive treatment, an attempt was made to reduce the dose of oral medications. This however proved ineffective, as the phantom pain increased. The positive blockage effect persisted for 4 weeks, which made it possible to qualify the patient for pulsed radiofrequency (PRF) treatment.

Ultrasound‐controlled PRF treatment of illiac and sciatic nerves was performed in sterile conditions. Local anesthesia at the needle guiding point was achieved by administration of 1% lidocaine. A 10‐mm length 20G Cossman needle with an electrode was inserted. After stimulation with 2 Hz 0.4 mV evoking stump muscle contraction, 50 Hz 04 mV stimulation was applied, which resulted in clearly perceivable numbness and pressure within the stump and amputated limb.

The PRF treatment was performed using a Cosman C4 apparatus (Cosman lmt). The 150‐s radiofrequency procedure was applied three times for each nerve at a temperature of 42°C. In each case, the procedure was terminated by administration of 5 ml 1% lidocaine and 4 mg dexamethasone. Complete resolution of both the stump and phantom pain was achieved. This allowed discontinuation of the oral treatment. No recurrence of the symptoms was reported after 2 weeks, 3 months, and 6 months. One year after the procedure, the patient reported with stump pain and phantom pain estimated at 4‐5 points on the VAS scale. The procedure described above was repeated, which resulted in resolution of the symptoms for a year.

## 2 DISCUSSION

In the presence of concomitant stump and phantom pain, neuroma in the stump can be suspected and plastic surgery should be considered. It results in mitigation or resolution of stump pain but does not always reduce the phantom pain of the amputated limb.

Pain sensations meet the criteria for neuropathic numbness‐related, burning, and shooting pain and may have diverse locations. The basic treatment involves pharmacotherapy with antidepressants, anticonvulsants, and opioids.

Continuous radiofrequency (CRF) can cause damage to peripheral nerves with subsequent intensification of neuropathic pain ^4^. Pulsed radiofrequency (PRF) is a safer alternative therapy. This procedure consists in heating tissues to a temperature of 42°C and generation of a strong electromagnetic field, which changes nerve conduction ^5^, ^6^.

Radiofrequency procedures are applied in the treatment of many pain syndromes, especially in neuropathies ^6^, ^7^. Many centers apply these procedures to manage sacral pain. Publications on this issue have been collectively reviewed ^8^, ^9^, ^10^, ^11^, demonstrating the effectiveness of this method. However, meta‐analyses have not indicated clearly higher effectiveness of either CRF or PRF.

Reports on the treatment of joint pain and other syndromes contain information on the application of PRF in these cases ^12^, ^13^, ^14^, ^15^, ^16^, ^17^. Hence, this method was chosen in the case of the patient described in this report. Literature data on the invasive treatment of phantom pain syndrome confirm its effectiveness ^18^, ^19^, ^20^.

Since the pain was alleviated for a year, there was no need to introduce other more invasive treatments, for example DRG stimulation, in the case of the patient described ^21^.

## 3 CONCLUSION

Pulse radiofrequency is a safe method of fighting stung and phantom pain. It creates the possibility of treating cases that have exhausted other therapeutic options. Phantom pain (PhP) is reported by patients after limb amputation due to trauma or chronic diseases. We report the case of successful PhP treatment with pulsed radiofrequency (PRF) of the femoral and sciatic nerve. (PRF) reduced the intensity of pain for one year, improving the quality of life of the patient.

## CONFLICT OF INTEREST

None declared.

## AUTHOR CONTRIBUTIONS

Krzysztof Brzezinski, Anna R. Rękas‐Dudziak, Maruszewska Agnieszka: contributed to the design and implementation of the research, to the analysis of the results and to the writing of the manuscript. Krzysztof Brzeziński: performed the treatment with consulting with Anna R. Rękas and Maruszewska Agnieszka.
